# Supplementary material for: LncRNA DILA1 inhibits Cyclin D1 degradation and contributes to tamoxifen resistance in breast cancer
Source: Nat Commun. 2020 Nov 2;11:5513. doi: 10.1038/s41467-020-19349-w (PMC7608661; doi:10.1038/s41467-020-19349-w)

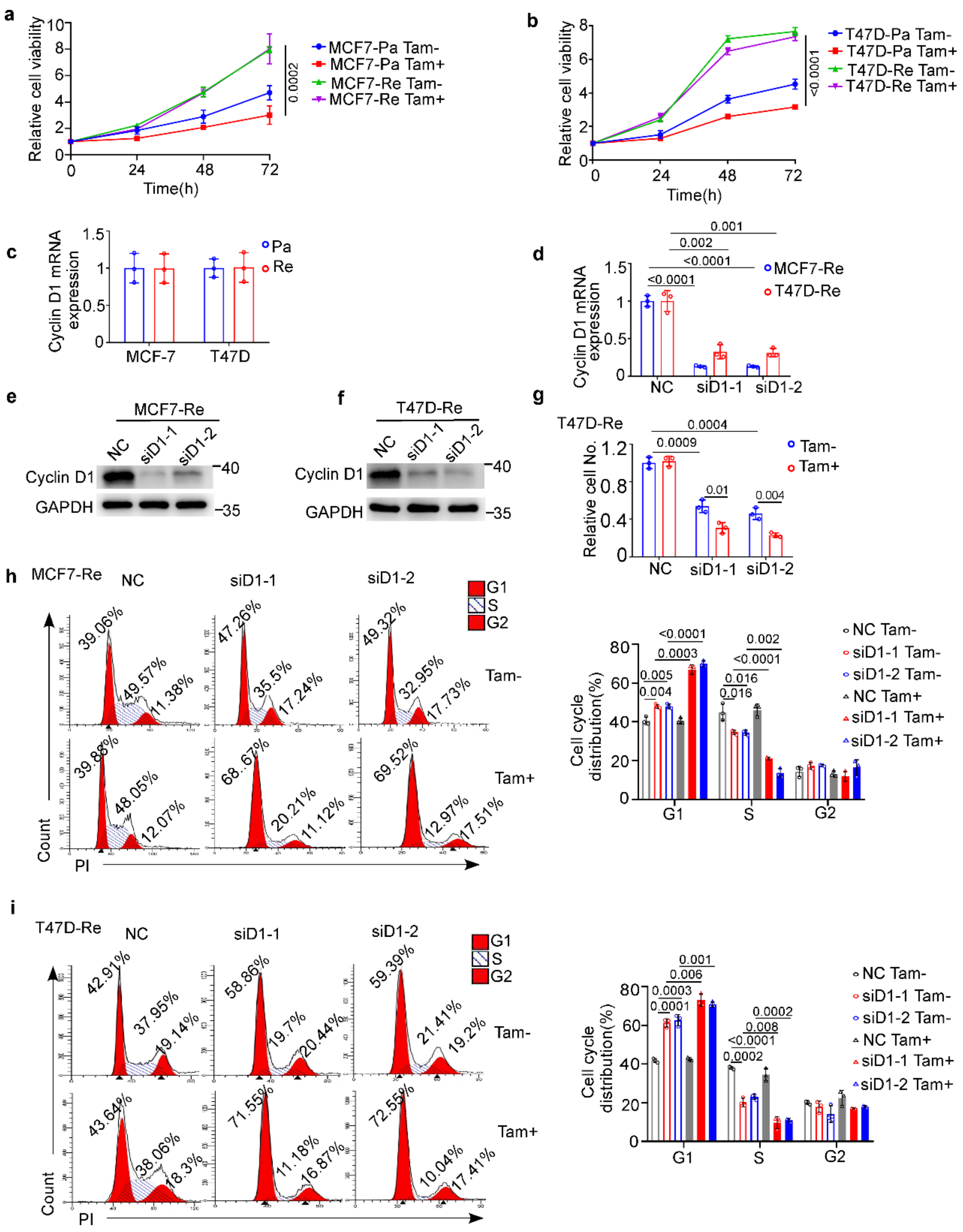

Figure S1. Upregulated Cyclin D1 is responsible for tamoxifen resistance in breast cancer cells

a and b The growth curves of parental and tamoxifen-resistant MCF-7 (**a**) or T47D (**b**) cells with or without tamoxifen(3μM) treatment, measured by MTT assay.

c RT-qPCR showing the expression of Cyclin D1 mRNA in parental and tamoxifen-resistant MCF-7 and T47D cells.

d RT-qPCR showing the expression of Cyclin D1 mRNA in MCF7-Re and

T47D-Re cells transfected with NC or siRNAs targeting Cyclin D1.

e and f Western blotting showing the expression of Cyclin D1 protein in MCF7-Re(e) and T47D-Re cells(f) transfected with NC or siRNAs targeting Cyclin D1. GAPDH as a loading control.

g T47D-Re cells, transfected with negative control siRNA (NC) or one of the two siRNAs targeting Cyclin D1 (siD1-1 and siD1-2), were treated with tamoxifen for 48hrs. Relative cell numbers were determined by a cell counter.

h, **i** Flow cytometry showing the cell cycle distribution of MCF7-Re (**h**) and T47D-Re (**i**) cells transfected with NC or siRNAs targeting Cyclin D1 and then treated with or without tamoxifen(3μM) for 48hrs. Representative images at the left panel and statistical analysis at the right panel.

For **a-i**, n = 3 biologically independent experiments. For **a–d**, **g-i**, means ± s.d. are shown, and p values were determined by two-tailed Students' test.

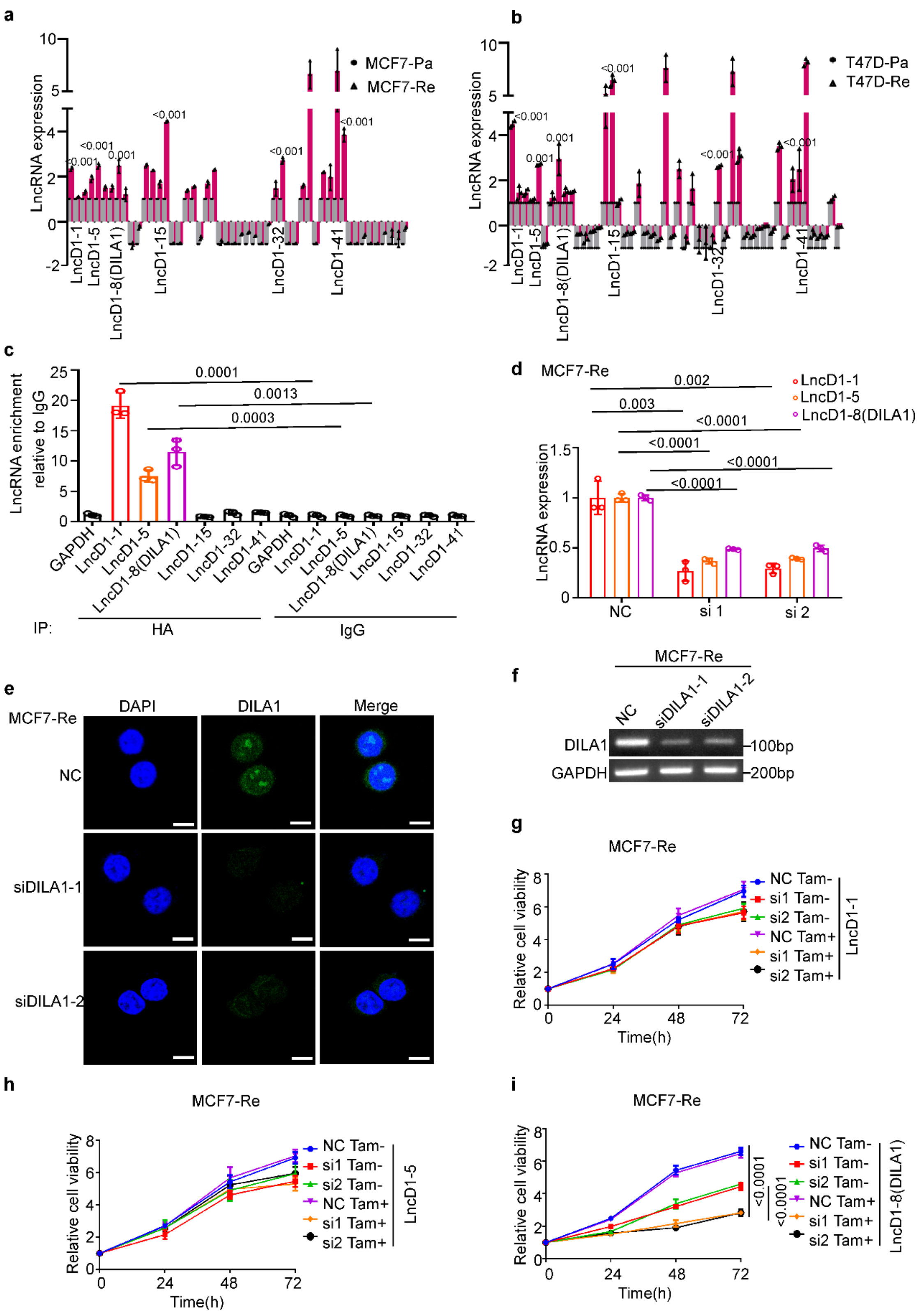

Figure S2. Identification of DILA1 among the 51 LncRNAs from RIP-Seq

a and b RT-qPCR showing the expression of 51 LncRNA identified by RIP-seq in MCF-7(**a**) and T47D(**b**) parental and tamoxifen-resistant cells.

c RIP-qPCR showing the six lncRNAs immunoprecipitated with anti-HA

antibody or IgG in MCF7-Re cells with ectopically expressed HA-Cyclin D1. GAPDH mRNA as a negative control.

d The Cyclin D1-interacting LncRNAs (LncD1-1, LncD1-5 and LncD1-8) were knocked down using respective siRNAs. RT-qPCR confirmed the efficiency of knockdown.

e Confocal FISH showing DILA1 expression in MCF7-Re cells transfected with NC or one of the two siRNAs targeting DILA1 for 48hrs. Representative images of three biologically independent experiments were shown, Scale bars represent 10 μm.

f Agarose gel electrophoresis(f) detected DILA1 expression in MCF7-Re cells transfected with NC or one of the two siRNAs targeting DILA1 for 48hrs. Representative images of three biologically independent experiments were shown.

g-i MTT assay showed the sensitivity to tamoxifen in MCF7-Re cells after the knockdown of LncD1-1(**g**), LncD1-5(**h**) and LncD1-8(**i**).

For **a-d**, **g-i,** n = 3 biologically independent experiments, means ± s.d. were shown, and p values were determined by two-tailed Students' test.

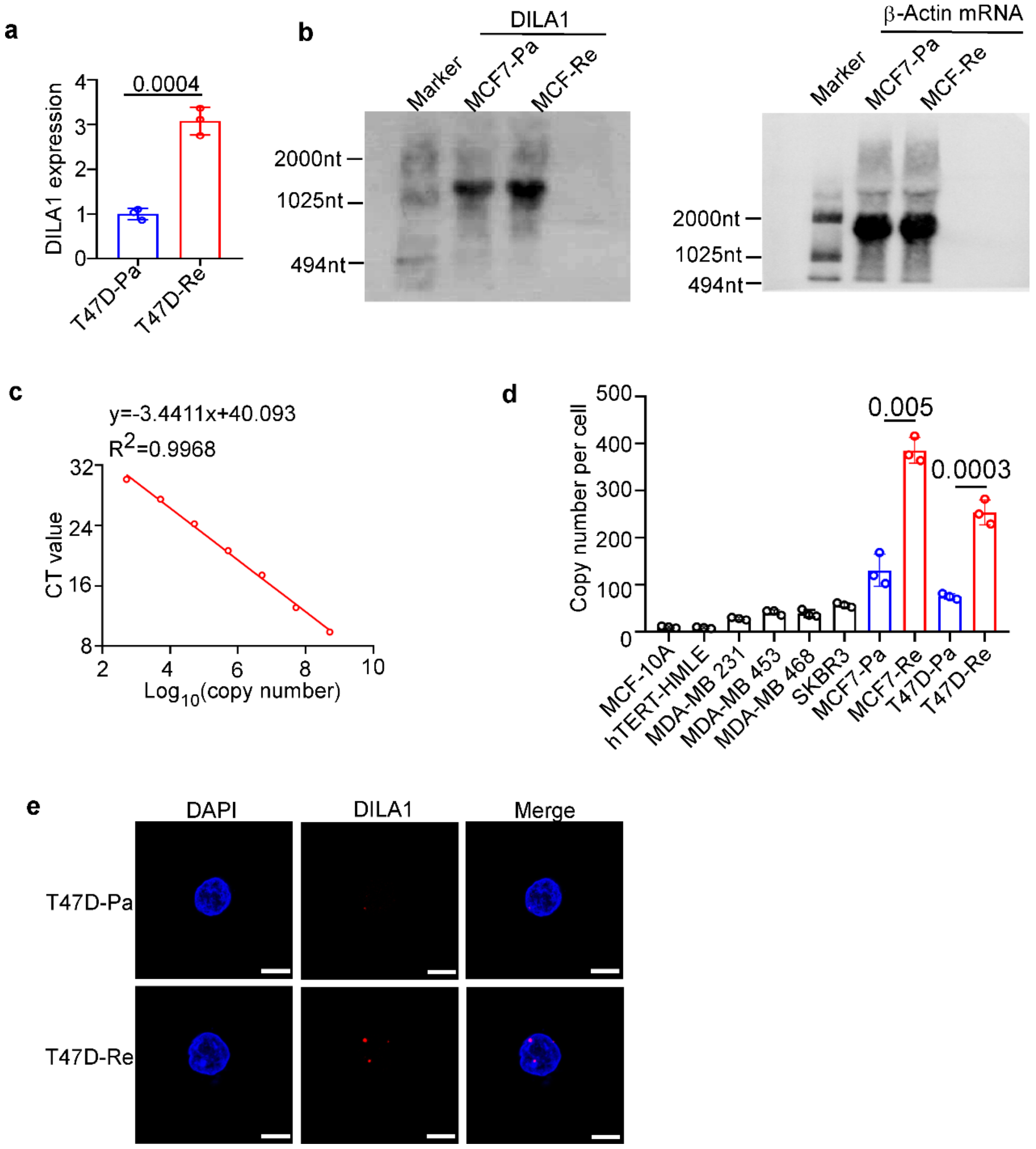

Figure S3 Supplement for figure 1, LncRNA DILA1 is overexpressed in tamoxifen resistant breast cancer cells

a RT-qPCR showing the expression of DILA1 in T47D-Pa and T47D-Re cell.

b Northern blotting showing the expression of DILA1(left panel) in MCF7-Pa and MCF-Re cells. β -Actin mRNA(right panel) as a loading control. Representative images of three biologically independent experiments were shown.

cThe standard curve for calculating DILA1 copy number.

d The copy numbers of two immortalized breast epithelial cell lines and 8 breast cancer cell lines.

For **a** and **d,** n = 3 biologically independent experiments, means ± s.d. were shown, and p values were determined by two-tailed Students' test.

e RNAScope showing subcellular localization and relative expression of DILA1(red) in T47D-Pa and T47D-Re cells. Representative images of three biologically independent experiments were shown, scale bars represented 10 μm.

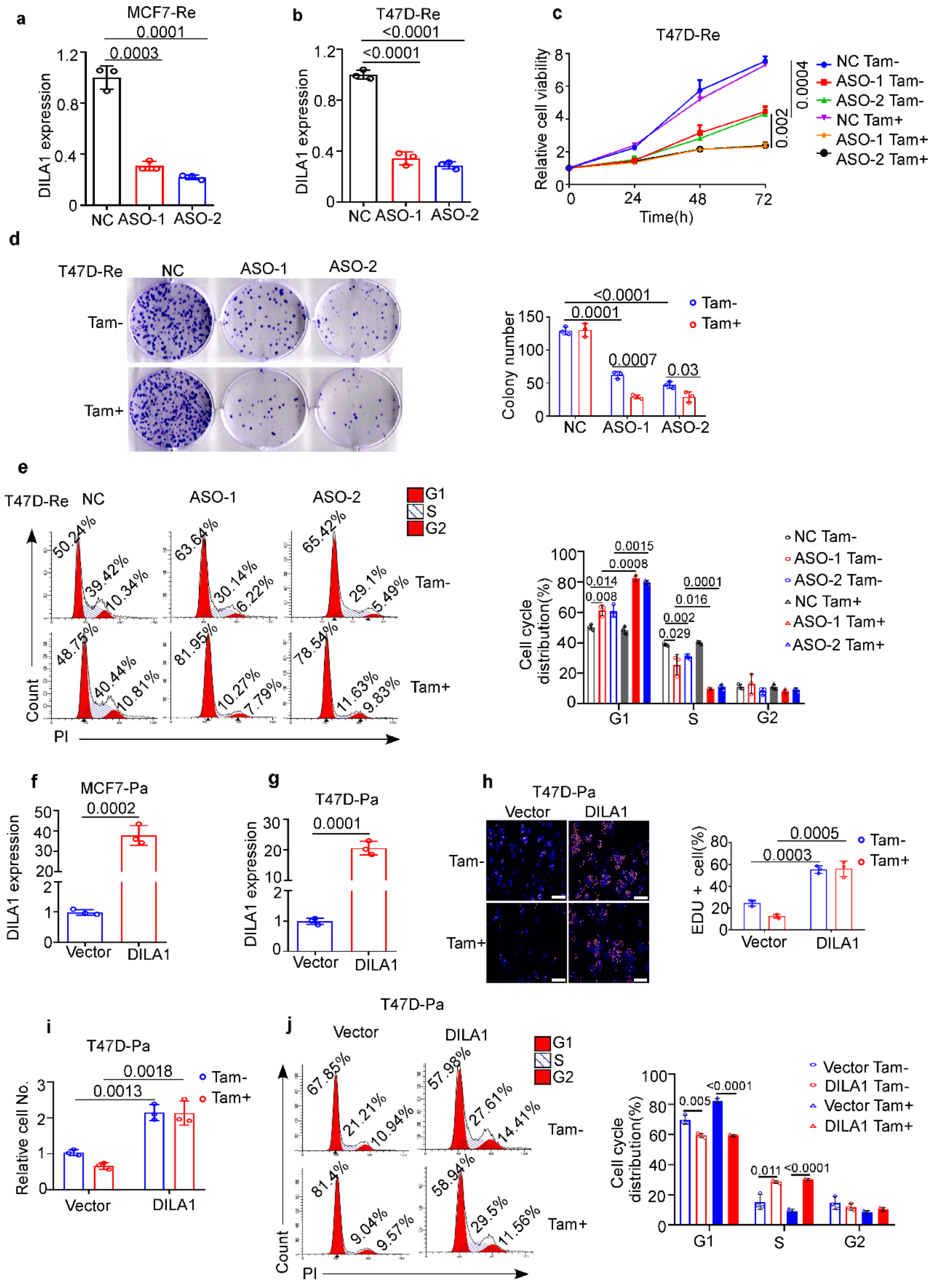

Figure S4. Supplement for figure 3, DILA1 promotes cell proliferation and tamoxifen resistance in MCF-7 and T47D cells.

a and b RT-qPCR showing the expression of DILA1 in MCF7-Re (**a**) and T47D-Re (**b**) cells transfected with NC or ASOs targeting Cyclin D1.

c T47D-Re cells were transfected with NC or ASOs targeting DILA1 and then

treated with 3μM tamoxifen (Tam). MTT assay showing relative cell growth at 0, 24, 48 and 72 hours(h).

d Representative images of colony formation.

e Flow cytometry showing the cell cycle distribution of cells.

Representative images at the left panel and statistical analysis at the right panel(**d, e**).

f and g RT-qPCR showing DILA1 overexpression in MCF7-Pa (**f**) and T47D-Pa (**g**) cells.

h - j T47D-Pa cells were transfected with control vector or vector expressing DILA1 and then treated with 3μM tamoxifen (Tam). Representative images of EdU incorporation by fluorescence microscopy(**h**). Relative cell numbers were determined by a cell counter(**i**). Flow cytometry showing the cell cycle distribution of cells(**j**). Representative images at the left panel and statistical analysis at the right panel (**h**, **j**). For **h,** scale bars represented 100 μm.

For **a-j**, n = 3 biologically independent experiments. Means ± s.d. were shown, and p values were determined by two-tailed Student's test.

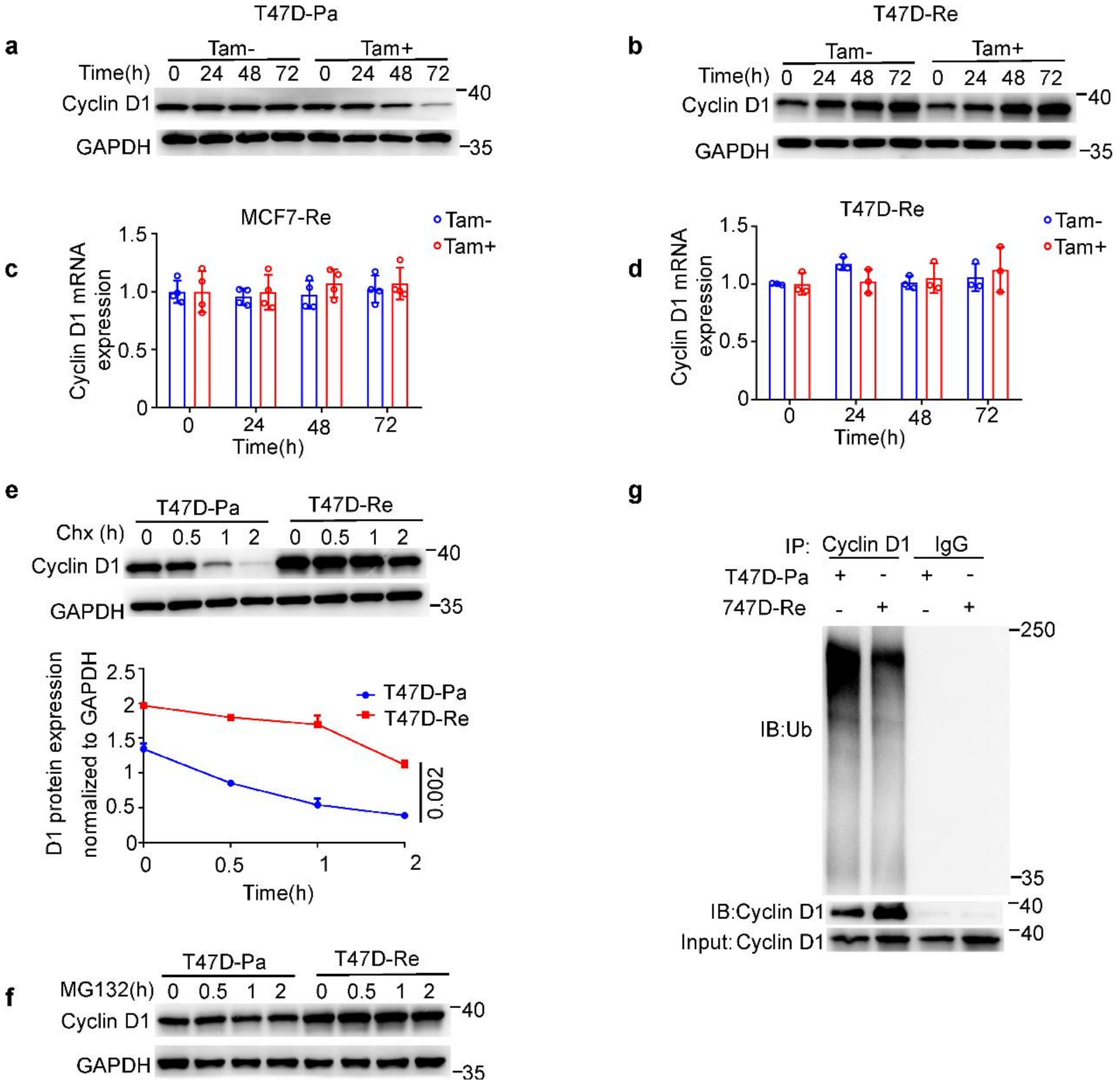

Figure S5. Supplement for Figure 3, DILA1 inhibits Cyclin D1 degradation via the ubiquitin-proteasome pathway.

a and **b** Western blotting showing Cyclin D1 protein at different time points after tamoxifen treatment in T47D-Pa (**a**) and T47D-Re (**b**) cells. GAPDH as a loading control.

c and **d** RT-qPCR showing the mRNA levels of Cyclin D1 at different time points after tamoxifen treatment in MCF7-Re (**c**) and T47D-Re (**d**) cells.

e Western blotting showing Cyclin D1 protein in T47D-Pa and T47D-Re cells treated with CHX for the indicated time. GAPDH was used as a loading control(top). The quantification of Cyclin D1 degradation rate was done by gray scale analysis(bottom).

For **c**, n=4 biologically independent experiments. For **d**,**e,** n = 3 biologically independent experiments. For **c-e**, means ± s.d. were shown, and p values

were determined by two-tailed student's test.

f Western blotting showing Cyclin D1 protein in T47D-Pa and T47D-Re cells treated with MG132 for the indicated time. GAPDH as a loading control.

g Ubiquitinated Cyclin D1 detected by immunoprecipitation with anti-Cyclin D1 antibody or IgG control and immunoblotting with anti-ubiquitin antibody in T47D-Pa and T47D-Re cells. For a, b, e-g, representative images of three biologically independent experiments were shown.

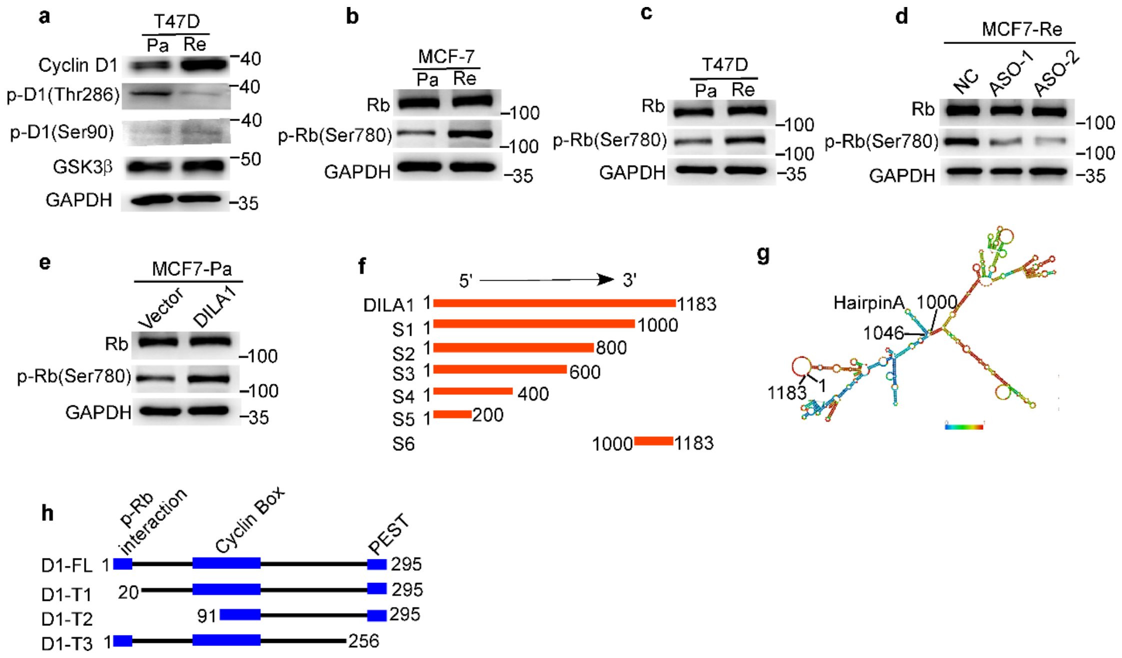

Figure S6. Supplement for figure 4, hairpin A of DILA1 interacts with Thr286 of Cyclin D1 and inhibits its phosphorylation.

a Western blotting showing the levels of Cyclin D1, p-D1 (Thr286), p-D1 (Ser-90) and GSK3β in T47D-Pa and T47D-Re cells.

b and c Western blotting showing the expression of Rb and p-Rb(Ser780) in parental and tamoxifen-resistant MCF-7(**b**) and T47D(**c**) cells.

d and e Western blotting showing the levels of Rb and p-Rb(Ser780) in MCF-Re cells transfected with NC or DILA1-ASOs (**d**), and in MCF7-Pa cells transfected with control vector or DILA1-overexpressed vector (**e**).For **a-e,** GAPDH as a loading control. Representative images of three biologically independent experiments were shown.

f, A schematic diagram of serial truncation mutants of DILA1 for RNA pull-down. **g**, The secondary structure of DILA1 predicted by RNAfold software. A stable hairpin structure was formed between 1000nt to 1183nt region of DILA1.

h, A schematic diagram of the Cyclin D1 truncation mutants, based on the three functional domains (“p-Rb interaction”, “Cyclin Box” and “PEST”).D1-FL: full length of Cyclin D1,1-295aa; D1-T1: truncation mutant 1 of Cyclin D1,20-295aa; D1-T2: truncation mutant 2 of Cyclin D1, 91-295aa; D1-T3: truncation mutant 1 of Cyclin D1,1-256aa.

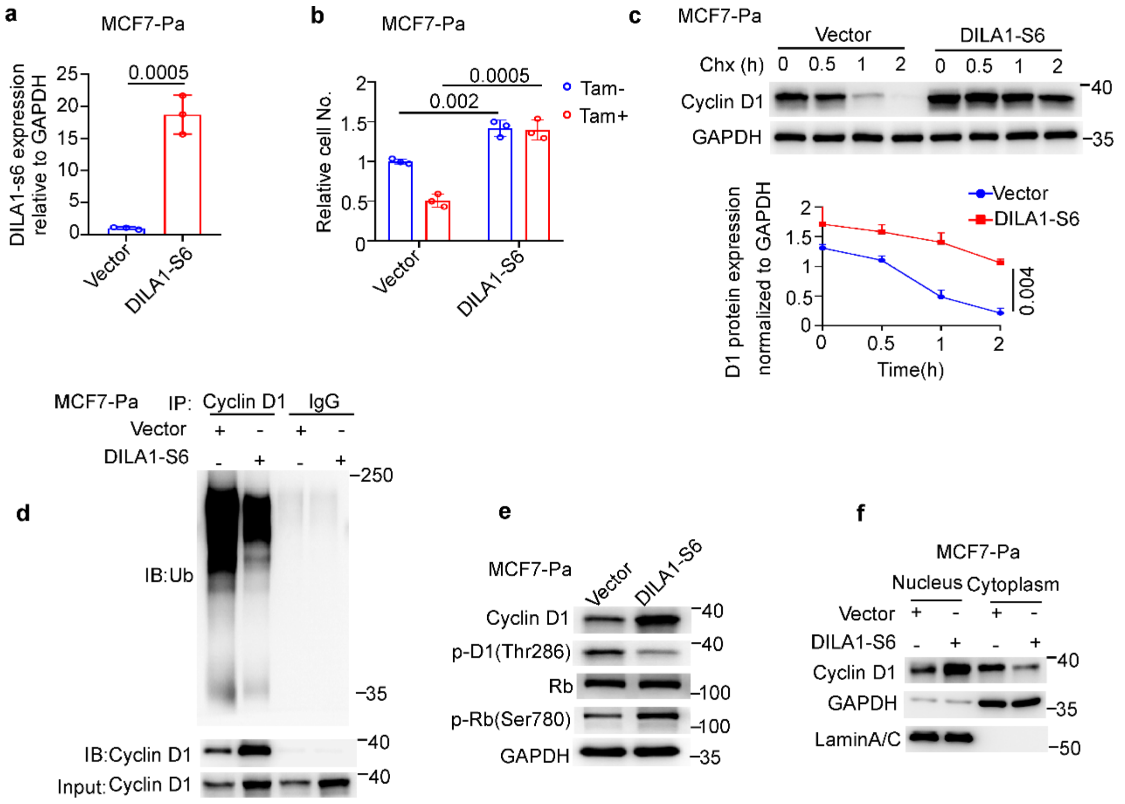

Figure S7. The truncated mutant of DILA1(1000-1183nt, DILA1-S6) inhibits the ubiquitin-proteasome degradation of Cyclin D1 via blocking the phosphorylation (Thr-286) of it.

a and **b** MCF-Pa cells were transfected with control vector(vector) or vector expressing DILA1-S6(DILA1-S6) and then treated with tamoxifen (Tam). (**a**) DILA1-S6 overexpressed in MCF7-Pa cells was confirmed by RT-qPCR. (**b**) Relative cell numbers were determined by a cell counter.

c Western blotting showing Cyclin D1 protein in MCF-7-Pa cells transfected with vector or DILA1-S6 for 48hrs and then treated with CHX for the indicated time (top). The quantification of Cyclin D1 degradation rate by gray scale analysis (bottom). For **a-c**, n = 3 biologically independent experiments. Means ± s.d. were shown, and p values were determined by two-tailed student's test.

d Ubiquitinated Cyclin D1 detected by immunoprecipitation with anti-Cyclin D1 antibody or IgG control and immunoblotting with anti-ubiquitin antibody in MCF-7-Pa cells transfected with vector or DILA1-S6 for 48hrs.

e Western blotting showing the levels of Cyclin D1, p-D1 (Thr286), Rb and p-Rb(Ser780) in MCF-7-Pa cells transfected with vector or DILA1-S6 for 48hrs. For **c** and **e**, GAPDH as a loading control.

f Western blotting showing the levels of nuclear and cytoplasmic Cyclin D1 in MCF-7-Pa cells transfected with vector or DILA1-S6 for 48hrs. GAPDH and Lamin A/C was used as cytoplasmic and nuclear control respectively. For **d-f**, representative images of three biologically independent experiments were shown.

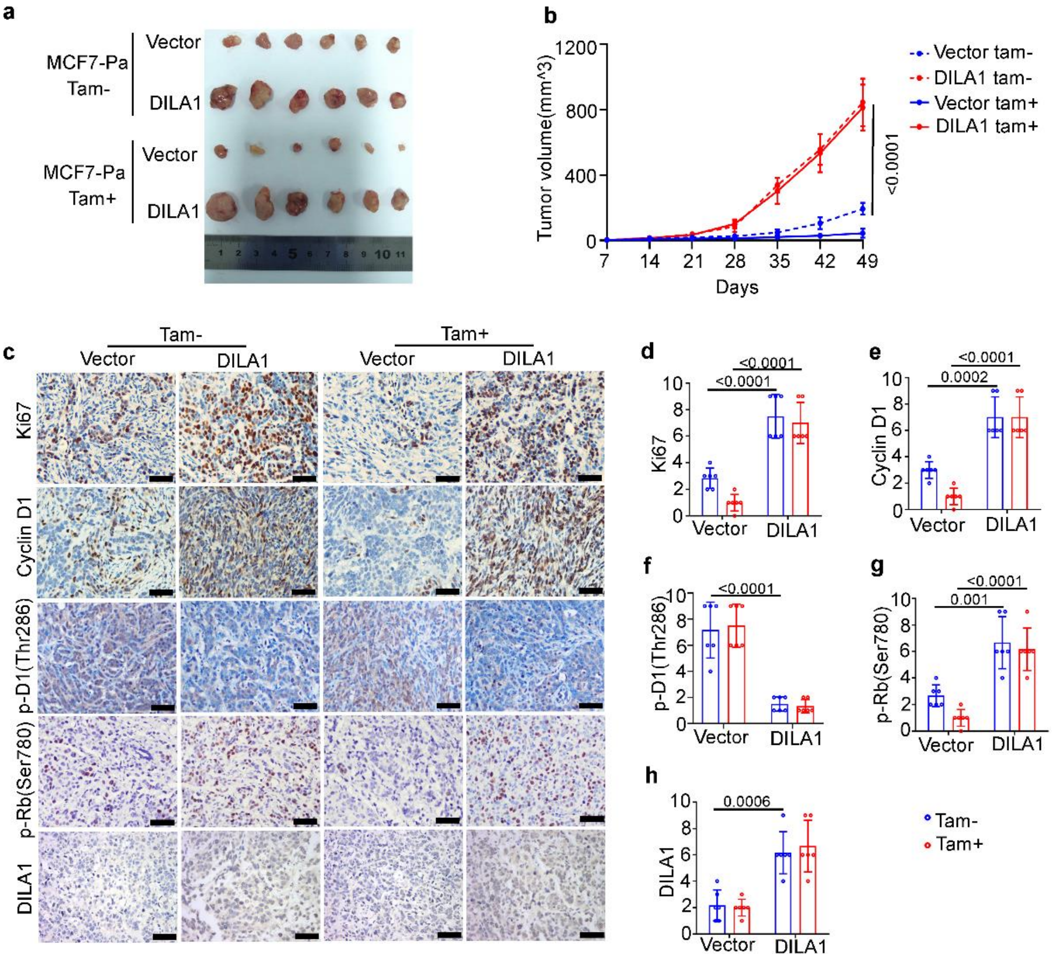

Figure S8. Supplement for Figure 5. DILA1 promotes tamoxifen resistance in vivo

MCF7-Pa cells with control vector or stable expression of DILA1 were inoculated into the mammary fat pads of NOD/SCID mice. **a and b** The tumor picture (**a**) and the tumor growth curve(**b**) were shown and compared among the groups.

c Immunohistochemistry (IHC) staining of Ki67, Cyclin D1, p-D1(Thr286) and p-Rb(Ser780) and In situ hybridization (ISH) staining of DILA1 in the tumors. Representative images of six xenografts from each group were shown.

d-h The IHC scores of Ki67(**d**), Cyclin D1(**e**), p-D1(Thr286) (**f**) and p-Rb(Ser780)(**g**) and ISH score of DILA1(**h**).

For **b, d-h,** n=6 mice per group, means ± s.d. were shown, and p values were determined by two-tailed Student’s test. Scale bar represented 50μm.

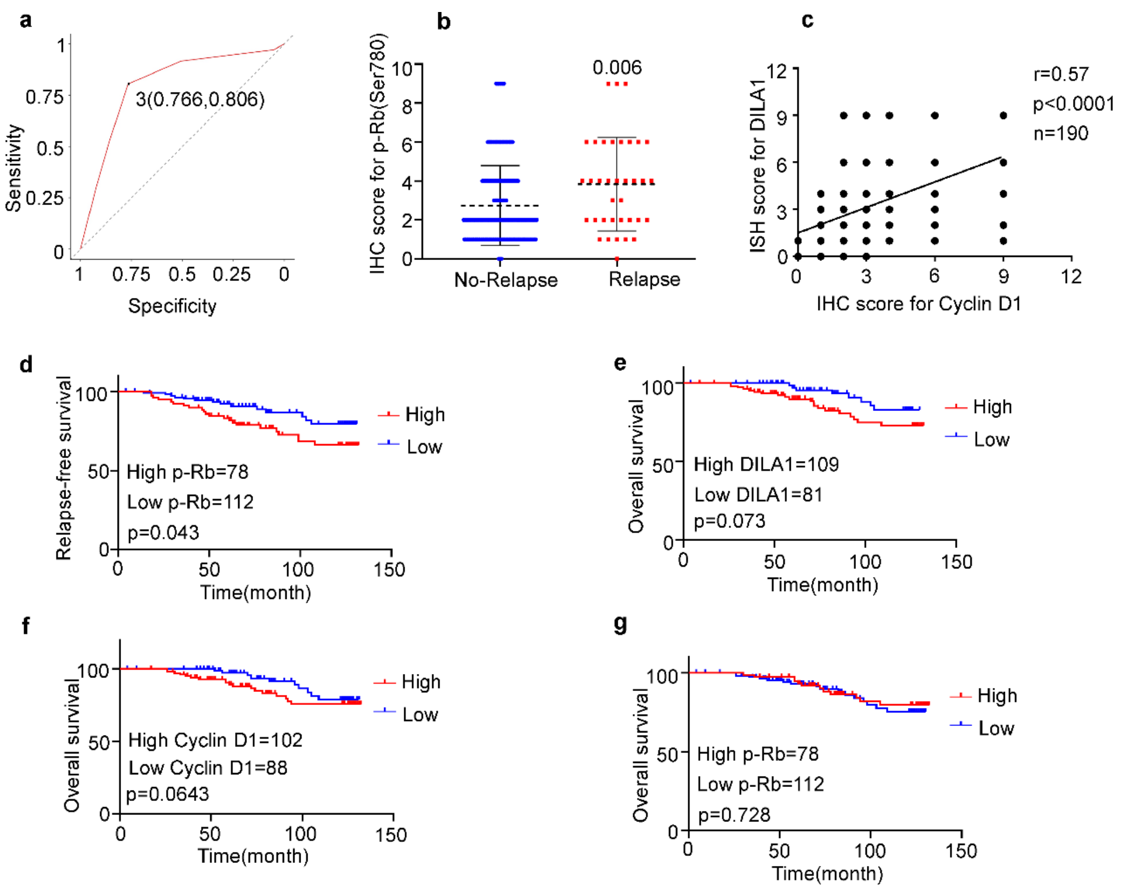

Figure S9. Supplement for Figure 6. High DILA1 expression is associated with higher Cyclin D1 and its downstream p-Rb protein expression, tamoxifen resistance and poor prognosis in ER positive breast cancer patients.

a ROC curve showing the cut-off value of high and low DILA1 expression.

b Staining index for p-Rb(Ser780) in the breast cancer specimens from ER positive patients with(n=36) or without relapse(n=154). n=190, means ± s.d. were shown, p values were determined by two-tailed Student's test.

c The correlation between DILA1 and Cyclin D1 in the breast cancer specimens from 190 ER positive breast cancer patients, determined by two-tailed spearman rank correlation analysis.

d Relapse-free survival of ER positive breast cancer patients was analyzed by Kaplan-Meier plots. Patients were divided based on the high or low expression of p-Rb(Ser780). n=190, p values were determined by two-tailed log-rank test.

e-g Overall survival of ER positive breast cancer patients was analyzed by Kaplan-Meier plots. Patients were divided based on the high or low expression

of DILA1(**e**), Cyclin D1(**f**) and p-Rb(Ser780)(**g**). For **d-g**, n=190, p values were determined by two-tailed log-rank test.

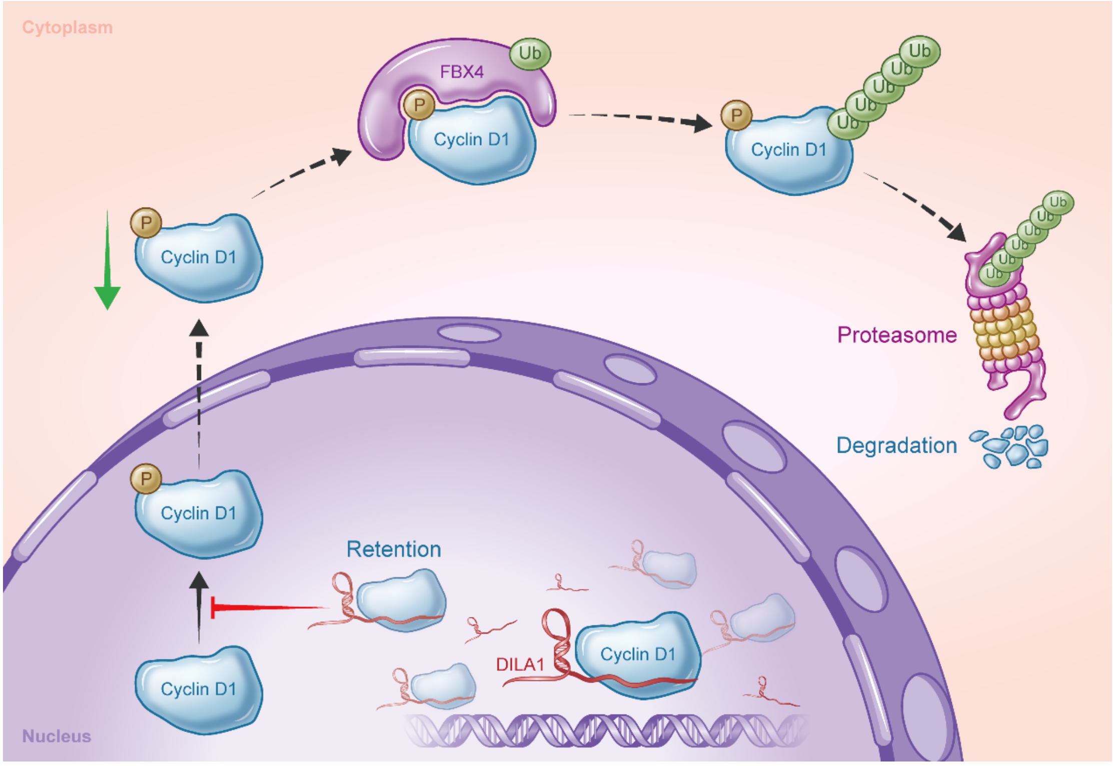

Figure S10 Schematic illustration of DILA1 inhibiting the ubiquitin-proteasome degradation of Cyclin D1 via blocking the phosphorylation (Thr-286) of it.

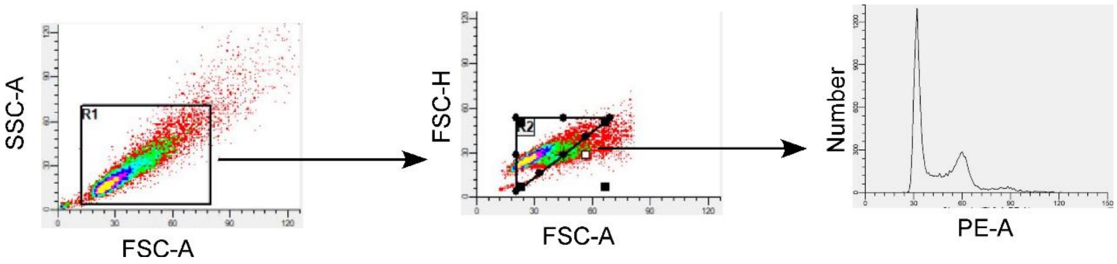

Figure S11 The gating strategy for flow cytometry to analyze cell cycle distribution

Table S1 The 51 LncRNAs enriched in RIP-Seq

Number	ncRNA	RNA type	Cyclin D1	HA-Cyclin D1	FC	log2(FC)	P value

LncD1-1	ENST00000607968.1	antisense	5.713	33.080	5.790	2.534	0.008
LncD1-2	ENST00000554678.1	lincRNA	2.449	25.729	10.51	3.393	0.005
LncD1-3	ENST00000568384.1	lincRNA	4.081	30.630	7.505	2.908	0.005
LncD1-4	ENST00000564287.1	lincRNA	6.530	34.305	5.254	2.393	0.009
LncD1-5	ENST00000606624.1	lincRNA	7.346	37.981	5.170	2.370	0.007
LncD1-6	ENST00000434309.1	lincRNA	25.302	85.763	3.390	1.761	0.002
LncD1-7	NR_110826.1	ncRNA	181.197	365.105	2.015	1.011	0.001
LncD1-8	ENST00000435697.1	lincRNA	51.421	123.744	2.406	1.267	0.006
LncD1-9	ENST00000442884.1	antisense	37.545	98.015	2.611	1.384	0.007
LncD1-10	ENST00000558342.1	antisense	13.875	57.584	4.150	2.053	0.003
LncD1-11	ENST00000609363.1	antisense	6.530	36.756	5.629	2.493	0.006
LncD1-12	NR_038951.1	ncRNA	0.816	15.927	19.514	4.286	0.016
LncD1-13	NR_038952.1	ncRNA	0.816	15.927	19.514	4.286	0.016
LncD1-14	NR_110018.1	ncRNA	2.449	19.603	8.006	3.001	0.024
LncD1-15	NR_104117.1	ncRNA	3.265	24.504	7.505	2.908	0.013
LncD1-16	NR_044997.1	ncRNA	4.081	20.828	5.104	2.352	0.048
LncD1-17	NR_119377.1	ncRNA	5.713	28.179	4.932	2.302	0.022
LncD1-18	NR_049793.1	ncRNA	6.530	28.179	4.316	2.110	0.032
LncD1-19	NR_136321.1	ncRNA	8.162	29.404	3.603	1.849	0.048
LncD1-20	NR_110174.1	ncRNA	12.243	41.656	3.402	1.767	0.024
LncD1-21	NR_134930.1	ncRNA	12.243	39.206	3.202	1.679	0.035
LncD1-22	NR_024418.1	ncRNA	20.405	61.259	3.002	1.586	0.014
LncD1-23	NR_134564.1	ncRNA	17.956	51.458	2.866	1.519	0.028
LncD1-24	NR_121682.1	ncRNA	23.670	67.385	2.847	1.509	0.014
LncD1-25	NR_120536.1	ncRNA	19.589	50.233	2.564	1.359	0.048
LncD1-26	NR_028308.1	ncRNA	22.854	56.358	2.466	1.302	0.044
LncD1-27	NR_047538.1	ncRNA	28.567	64.935	2.273	1.185	0.048
LncD1-28	NR_047537.1	ncRNA	39.178	84.538	2.158	1.110	0.037
LncD1-29	NR_036555.1	ncRNA	63.664	129.869	2.040	1.029	0.021
LncD1-30	ENST00000431150.1	lincRNA	0.816	13.477	16.512	4.045	0.033
LncD1-31	ENST00000451766.2	lincRNA	1.632	15.927	9.757	3.286	0.035
LncD1-32	ENST00000455105.1	lincRNA	1.632	14.702	9.006	3.171	0.048
LncD1-33	ENST00000585827.1	lincRNA	3.265	23.278	7.130	2.834	0.017
LncD1-34	ENST00000609583.1	lincRNA	4.897	24.504	5.004	2.323	0.032
LncD1-35	ENST00000423967.1	lincRNA	12.243	45.332	3.703	1.889	0.013
LncD1-36	ENST00000492208.1	lincRNA	10.611	35.530	3.349	1.744	0.038
LncD1-37	ENST00000607988.1	lincRNA	42.443	91.889	2.165	1.114	0.030
LncD1-38	ENST00000501068.2	lincRNA	36.729	78.412	2.135	1.094	0.046
LncD1-39	ENST00000606790.1	lincRNA	48.972	98.015	2.001	1.001	0.043
LncD1-40	NR_104331.1	ncRNA	14.692	42.881	2.919	1.545	0.040
LncD1-41	ENST00000441363.1	antisense	0.816	13.477	16.512	4.045	0.033
LncD1-42	ENST00000415166.1	antisense	0.816	12.252	15.011	3.908	0.048
LncD1-43	ENST00000585184.1	antisense	0.816	12.252	15.011	3.908	0.048

LncD1-44	ENST00000507091.1	antisense	4.081	24.504	6.004	2.586	0.021
LncD1-45	ENST00000608684.1	antisense	4.081	22.053	5.404	2.434	0.037
LncD1-46	ENST00000578482.1	antisense	4.081	20.828	5.104	2.352	0.048
LncD1-47	ENST00000354527.2	antisense	4.897	23.278	4.753	2.249	0.042
LncD1-48	ENST00000597609.1	antisense	6.530	29.404	4.503	2.171	0.025
LncD1-49	ENST00000504573.1	antisense	20.405	52.683	2.582	1.368	0.042
LncD1-50	ENST00000586560.1	antisense	22.854	58.809	2.573	1.364	0.033
LncD1-51	ENST00000520840.1	antisense	31.016	75.961	2.449	1.292	0.023

p values were determined by negative binomial generalized linear models. No adjustments were made for multiple comparisons.

Table S2 The Sequence of Full Length DILA1 (5’-3’)

AGCTTCTGCTAGTGTTTAGGTACTAGTGCAGTGTAGAACCCTTTTCTCAGGCAAAACCTAACATCTGACCGCA
CCCTTTTTGCTATTAAAAAAAAAAAAAAAGCTTCTATTTTTTTCCTCCTGTCGTTAAGATAAATTCTCCAGTAGACA
ACCTCTCCCACTAATTGTGTATTAGCAACGTATTTTATCTTGAGAGCTTGTACACTGGGCTCTCAACGTGGTGT
GGAAGGTAGCCTGTTACAGTGCTGGATTCATAAAAGGGCCTTTATGGTTTGTGAAAGAATATCTGTGTGCTTA
GGGAGGAAACTTTTTGATCTGCAGAAAAGCCAGAAGACATCTAGGACATCCATAAAAATTCATCAGAGAGCAT
TTTACTACTGAGCTGCAAAGGTAGGAAAAATAAATTTTTTTTGGTTAGAAAATATACTTCTTTCATCTGCGTTTCA
ATAATTTATTCCCCTTGTTAGATATACATGGAATTGATATTGCTTATTCTGACTAAGTGAAAAGTGTTATTTCTACT
TACTGCTCAATTAGAAACATGTAGTGTTTAATTGTCAGTTTGAATTTTTCTTTCATACTATTACAGTTTATATTTTA
CTATAGGCAGAATTTCCTCTTTATCCTAAATTTTGATATTCTCCATAATTTCATCGTGCCTTGACATTGCTGTATAT
AATTTCTATACATCAGGAAGGCAATTCTGTGTGCATGTGTGTGTGTATATATATAGTTAGAAGTTAGAAACTGAA
ACTCTTTTTTGTCTACCATCATTTCATATTTTTCTTGCTATTTCAAACCTATAATTTTTAAAGAAACGACTAACGTA
ATCATTCATAGAATATGCTGTCAGTAACTTAACAATATAAATGTTAACATGGAGGCATTTAGTTCAGCATTTGCTT
TACTTAATAGAGTGCAGAAAATGTGGCCCTTTTCTCAGTTAGGGTGTATGTGAAGATAAATTTAGAATTTCTAGA
CTAGTAAATACTTCTCTGATAATAACTTAATTATAGCTATTTCAAACCAAATTGTTTAATTGTGGTGAATCATATGT
AAATTGTTCAGATGTAGGTCAACACCCACGCCCACATTCTAGCACCTCTTTCCTAATTCAAGACAACTTTGCAG
TGCCTAAAAGAGCTGAACAGTGCAGATTAGAGACATAAGACAAAAAAAAAAAA

Table S3 Sequences for ASOs, siRNAs and primers used in this study

**Sequences for ASOs or siRNAs**	**5'-3'**
ASO-1	GACAACTTTGCAGTGCCTAA
ASO-2	GTCAACACCCACGCCCACAT
sicyclinD1-1	CCAAUAGGUGUAGGAAAUAGCGCTG
sicyclinD1-2	GCCCTCGGTGTCCTACTTCAA
siLnc-624-1	GUCAAGAGAUGAAAGACAATT
siLnc624-2	CAGUAUUAUCGAAGAUCAATT

siLnc968-1	UCUUAGUUAUCUACUGUUATT	
siLnc968-2	GGCUCACGAUACAUAUAUATT	
siDILA1-1	GGGCCUUUAUGGUUUGUGATT	
siDILA1-2	GACAACCUCUCCCACUAAUTT	
**Primers for RACE**	**5'-3'**	
5'RACE primer1	TACAAGCTCTCAAGATAAAATACG	
5'RACE primer2	AATACGTTGCTAATACACAATTAG	
3'RACE primer1	TACTTCTTTCATCTGCGTTTCAAT	
3'RACE primer2	TGTTATTTCTACTTACTGCTCAAT	
**Primers for RT-qPCR**	**5'-3'**	
Name	Forward	Reverse
GAPDH	GGAGCGAGATCCCTCCAAAAT	GCTGTTGTCATACTTCTCATGGG
MALAT1	GACGGAGGTTGAGATGAAGC	ATTCGGGGCTCTGTAGTCCT
DILA1	CCTGTTACAGTGCTGGATTCAT	TGTCC TAGATGTCTTCTGGCTT
Cyclin D1	GCTGCGAAGTGGAAACCATC	CCTCCTTCTGCACA CATTTGAA
ENST00000607968.1	GTATGCCCTGCCACATTTCT	TCAGCGATGGTTGAAACAAG
ENST00000609363.1	GATCTGAGGACCGAGTCCAG	CCAACGGTCTCAGGAATGTT
ENST00000558342.1	CAATGCCTGCTCTCAGACAA	TGCTTCCCACAAATTTTGATT
ENST00000442884.1	TCAGAGACATCCCTGGTTCC	TCTCCTTCCCCAGAATCCTT
ENST00000554678.1	TGAGGCATGAGAATTGCTTG	CTGATTTCAGGGAAGGGTGA
ENST00000568384.1	GAGGGACACCTGTGAAGGAA	ATGGCCTGTGTTGTGAGTGA
ENST00000564287.1	ACATTTCACAGCCCACCTTC	ACTGATGAGGACGGAAATCG
ENST00000606624.1	GGACGGCTCCCTTATAAACC	ATATCGAAAGTCCCGTGCTG
ENST00000434309.1	AGCAGAGGGAACAGAGACCA	GTTGCTCCCTTGCTTGAGAC
NR_110826.1	CCTTACAGGCTTGGAAGTGC	CTCCCAGGCTCAGCTATCAC
NR_038951.1	TTATTCTCCATGAATCATCA	CCAGGCAGAAACACAAAAGA
NR_038952.1	TCACCCAGGTTGCTAAGGGC	TCATTGAACAGGTCTCCTCT
NR_110018.1	CAGCAAAAGATATAATGGGC	TCCTTTCATCTTCAGGCTGC
NR_104117.1	GTCTGATGTTTACTGAAATC	AGTTTCAGGGCTCCATTAAA
NR_044997.1	CTAAGTTCAAACGAGG	GCCCAACAGGATAAGC
NR_119377.1	CACTCCCTATGGCTT	CCATTAACTTTCTTCTTC
NR_049793.1	CTGAACCCCGCGCCAGGAC	GCAGGACCAGCCCGACGGG
NR_136321.1	ACGCTGATGCCGACCCTG	CTTCCCAGGGGTGAGCCTC
NR_110174.1	CACCGGTTAAAACGCAG	GGTCCTAGCTACTTGGGAG
NR_134930.1	AGGAGGGCGCAGCATTG	GAGCAGTCCAGGACACGG

NR_024418.1	GTCTCCTGCTCAAAACAG	GGGAGTCCTCCGCGTCCTC
NR_134564.1	CTGACTGCGTGAACTCG	GCGAGGGATCCGACCG
NR_121682.1	GAGCAGCGCTCCCGCGC	CCGCAGCCCAGCCCCC
NR_120536.1	CACGGCCCTACCAGGAAC	GTTTGAAGAATGGAAG
NR_028308.1	CGGGGGCCTTCTCCATAG	CTGGTGCGGGGAGGTTG
NR_047538.1	CAAGCGAAATTAAACTAAACC	GACACTAGTCCGGCGCCAG
NR_047537.1	CTCGTCGAAAGTCTTCC	GCGGCTTTTAGTTCAAAAC
NR_036555.1	CCTAAATTCAAAAGCCAG	CTGGGTCAGCCCTTCGTTTC
ENST00000431150.1	CCTGAGGCCTGAAGATGG	CTCAGAATCGCTTTCCCC
ENST00000451766.2	CGAGAGGTCGTCGCCTTG	GAGCCGAGATCGCACCAC
ENST00000455105.1	CAGGACGACTGAGCAAG	GGCGTTTGGGGCTCTTTC
ENST00000585827.1	GCTGGTCGCAGGGCTGCG	CTTAGGAAAGCAGGAGG
ENST00000609583.1	GTAACTAACCAAATTACC	GGAACCCAGCGTCCATC
ENST00000423967.1	CGTATACCTCTGGAATAGC	GGATACAAAATCAGTCTCC
ENST00000492208.1	GGCAGAGTTTGGCTGCTGT	CCCCAGATGGCAACTTG
ENST00000607988.1	GTGATAAGCCTCTTACC	CCTCAGCCTCTCATAGTG
ENST00000501068.2	CGGGCGCTGCCGCCGGAAG	CCTAGCAGGCACAAGGG
ENST00000606790.1	GGGAACGTGCAGGGGGGC	GGACCGAGCTATTTAGAG
NR_104331.1	CCCAAGACAGGTCAGAG	CCCAGTCTCGAGGCCCAC
ENST00000441363.1	TCTGACCGTTCTCTGGC	CTGCTCACGGAACAGTG
ENST00000415166.1	GGATTCTGACCCATGG	CGGCCAGCGCTGGTAGTTTG
ENST00000585184.1	GCGGCCGCCCCAAGCTGAG	CTTACTACAAATGCAAATG
ENST00000507091.1	GAGAATTCAGCTGCTC	GAGACCAAGAACCCACC
ENST00000608684.1	GCTATTTGTCTGTTAAG	GGAATTTTGAAGACGAC
ENST00000578482.1	GTAACTCCATTGTTTTTC	CAGCAGCCGCAGCCGCC
ENST00000354527.2	GATCCCCGACATGCTGGC	GCTGCCGCCGCCGCCGC
ENST00000597609.1	CTGTATTTTATTGGAAAAC	CGGAATGTTTTGGTTTTG
ENST00000504573.1	CGGCCTGGGGGTCGTAC	CGGTTTTCCCCATGTGCCC
ENST00000586560.1	GGATTCAGACACAGAAC	CATGGGCCAGTGCCTGG
ENST00000520840.1	GGCATTAAATGAGCTCTC	GGAGGCTGTGGATGGCTG
**Primers for eCLIP-qPCR**	**Forward (5’-3’)**	**Reverse (5’-3’)**
Primer 1	TAGGTACTAGTGCAGTGTAGAA	TACTGGAGAATTTATCTTAACGA
Primer 2	TCCTGTCGTTAAGATAAATTC	CTACCTTCCACACCACGTTGAG
Primer 3	GTAGCCTGTTACAGTGCTGGA	GATGTCCTAGATGTCTTCT
Primer 4	GCCAGAAGACATCTAGGACATC	TCAATTCCATGTATATCTAAC
Primer 5	TACTTCTTTCATCTGCGTTTCA	CATGTTTCTAATTGAGCAGT
Primer 6	CATGTAGTGTTTAATTGTCAG	GATGAAATTATGGAGAATATC
Primer 7	ATCGTGCCTTGACATTGCTGTA	AGTTTCAGTTTCTAACTTCTA
Primer 8	ATAGTTAGAAGTTAGAAACTG	TAAGTTACTGACAGCATATTC
Primer 9	ATCATTCATAGAATATGCTGTC	TATCTTCACATACACCCTAAC
Primer 10	GCTTTACTTAATAGAGTGCAG	TCAGAGAAGTATTTACTAGTCT

Primer 11	ATAACTTAATTATAGCTATTTC	CTGCAAAGTTGTCTTGAATTA

Table S4 Correlation between DILA1 expression and Cyclin D1, p-D1 and p-Rb expression in 190 breast cancer patients

	DILA1(high) n=109	DILA1(low) n=81	P value
Cyclin D1			
High	80(73.4%)	22(24.7%)	<0.001
Low	29(26.6%)	59(72.8%)	
p-D1(Thr286)			
High	58(53.2%)	63(77.8%)	<0.001
Low	51(46.8%)	18(22.2%)	
p-Rb(Ser780)			
High	55(50.5%)	23(28.4%)	<0.005
Low	54(49.5%)	58(71.6%)	
Ki67			
High	74(67.9%)	31(38.3%)	<0.001
Low	35(32.1%)	50(61.7%)	

Table S4 Correlation between DILA1 expression and Cyclin D1, p-D1(Thr286), p-Rb(Ser780) and Ki67 expression in 190 ER positive breast cancer patients. ISH staining scores of DILA1 and IHC staining scores of Cyclin D1, p-D1(Thr286), p-Rb(Ser780) and Ki67 were calculated as the description in the method section. p values were determined by two-tailed Chi-square test.

Table S5 Correlation between DILA1 expression and clinical parameters in 190 breast cancer patients

Characteristics	DILA1-low n=81	DILA1-high n=109	P value
Age			
<59	61(75.3%)	85(77.9%)	0.665
>=59	20(24.7%)	24(22.1%)	
Tumor size			
<=T1	35(43.2%)	43(39.4%)	0.619
>T1	46(56.8%)	66(60.6%)	
LN metastasis			
<=N1	66(81.5%)	73(66.9%)	<0.05
>N1	15(18.5%)	36(33.1%)	
Clinical stage			
I,II	65(80.3%)	70(64.2%)	<0.05
III,IV	16(19.7%)	39(35.8%)	
Her-2			
-	43(53.1%)	61(55.9%)	0.693
+	38(46.9%)	48(44.1%)	
Ki67			
<=14%	51(62.9%)	35(32.1%)	<0.001
>14%	30(37.1%)	74(67.9%)	

Table S5 Correlation between DILA1 expression and clinical parameters in 190 ER positive breast cancer patients. All patients were divided into DILA1 low (SI<3, n=81) and DILA1 high (SI>=3, n=109) group, clinical variates included patient age, tumor size, LN (lymph node) metastasis, clinical stage and the expression of Her-2 and Ki67 were divided into categorical data, and the frequency was calculated. p values were determined by two-tailed Chi-square test.

Table S6 Correlation between Cyclin D1 expression and clinical parameters in 190 breast cancer patients

Characteristics	Cyclin D1-low n=88	Cyclin D1-high n=102	P value
Age			
<59	71(80.7%)	75(%)	0.351
>=59	17(19.3%)	27(%)	
Tumor size			
<=T1	37(42.0%)	41(%)	0.796
>T1	51(56.0%)	61(%)	
LN metastasis			
<=N1	68(77.3%)	71(%)	0.235
>N1	20(22.7%)	31%)	
Clinical stage			
I,II	66(75.0%)	69(%)	0.265
III,IV	22(25.0%)	33(%)	
Her-2			
-	47(53.4%)	57(%)	0.732
+	41(46.6%)	45(%)	
Ki67			
<=14%	47(53.4%)	38(37.3%)	<0.01
>14%	41(46.6%)	64(62.7%)	

Table S6 Correlation between Cyclin D1 expression and clinical characteristics in 190 breast cancer patients. All patients were divided into Cyclin D1 low (SI<3, n=88) and Cyclin D1 high (SI>=3, n=102) group, clinical variates included patient age, tumor size, LN (lymph node) metastasis, clinical stage and the expression of Her-2 and Ki67 were divided into categorical data, and the frequency was calculated. p values were determined by two-tailed Chi-square test.

Table S7 Effect of variates on relapse-free survival in 190 breast cancer patients in univariate and multivariate proportional hazards Cox regression model

Variates	Univariate		Multivariate	
	HR(95%CI)	P value	HR(95%CI)	P value
Age	1.189(0.578-2.448)	0.638	-	-
Tumor size	1.079(0.556-2.093)	0.823	-	-
LN metastasis	5.991(2.994-11.988)	<0.001	2.751(0.371-20.401)	0.322
Clinical stage	5.507(2.752-11.018)	<0.001	1.906(0.246-14.789)	0.537
Her-2	2.116(1.039-4.306)	0.039	-	-
Ki67	1.972(1.000-3.886)	0.050	-	-
DILA1	10.522(3.223-34.352)	<0.001	6.414(1.917-21.467)	<0.001
Cyclin D1	3.855(1.689-8.802)	0.001	2.287(0.984-5.316)	0.055

Table S7 Effect of variates on relapse-free survival in 190 breast cancer patients in univariate and multivariate Cox proportional hazards regression model. Variates included patient age, tumor size, LN (lymph node) metastasis, clinical stage and the expression of Her-2, Ki67, DILA1 and Cyclin D1 were analyzed in univariate Cox regression model, and the p values were determined by univariate Cox regression analysis. For the multiple Cox regression analysis, patient age, tumor size, Her-2 and Ki67 status were excluded due to the p value larger than 0.01. The variates with a p value smaller than 0.01 in univariate analysis were included in the multiple Cox regression model, and the p values were determined by multivariate Cox regression analysis, no adjustments were made for multiple comparisons.

Supplementary Figure for uncropped blots and gels

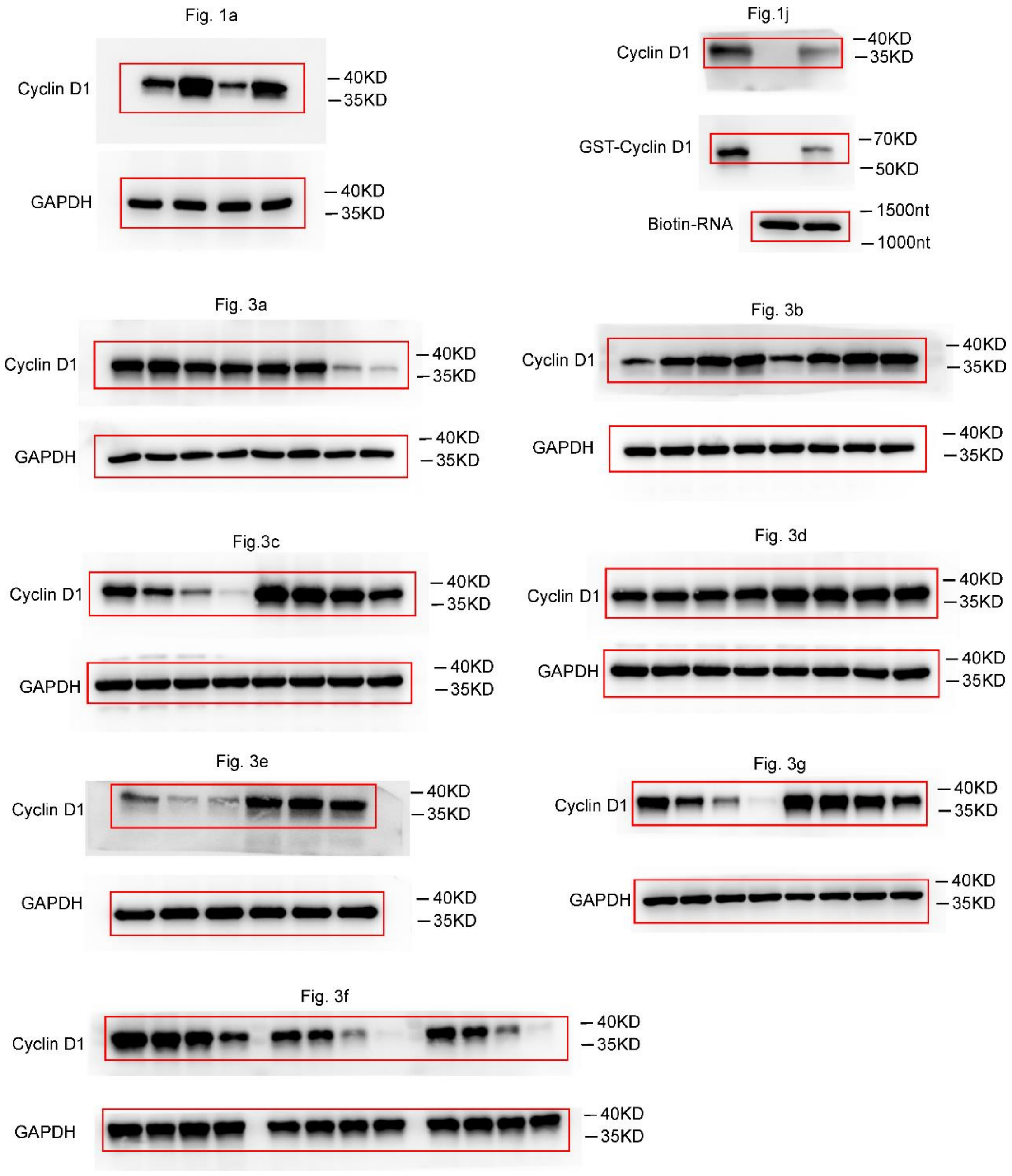

Fig. 3h

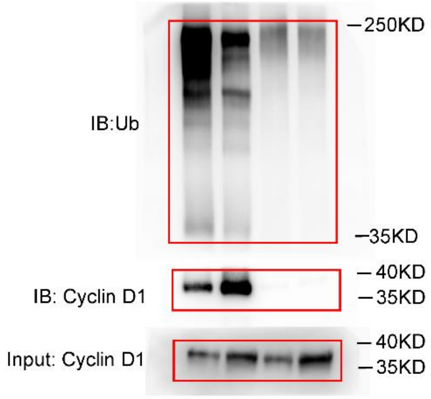

Fig. 3i

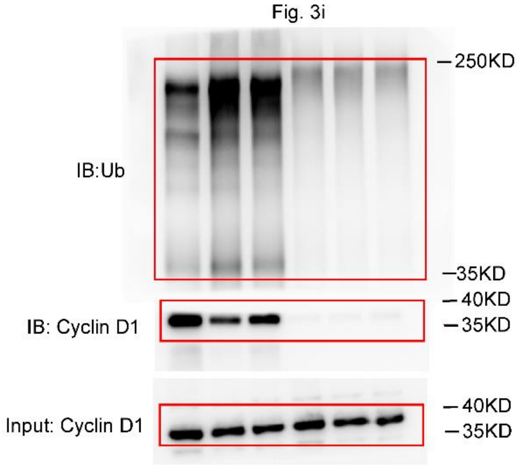

Fig. 4a

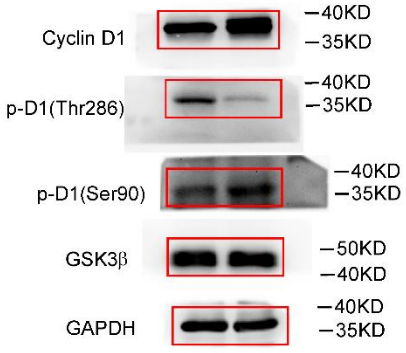

Fig. 4b

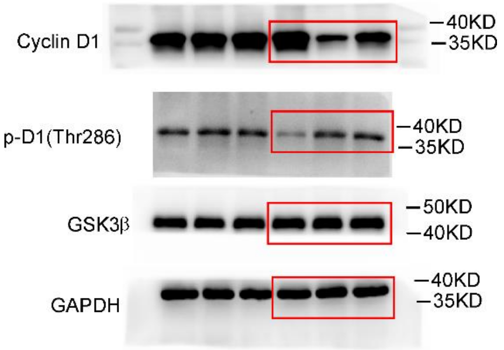

Fig. 4c

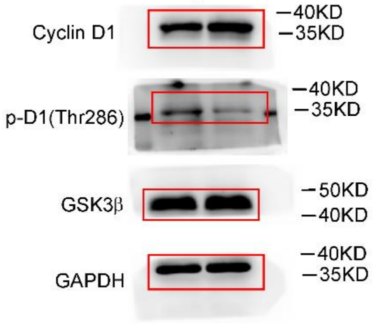

Fig. 4d

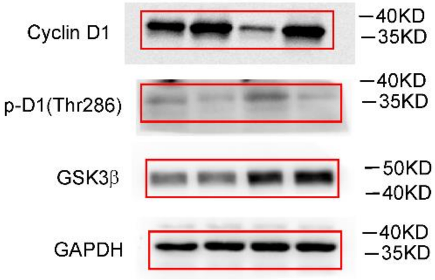

Fig. 4e

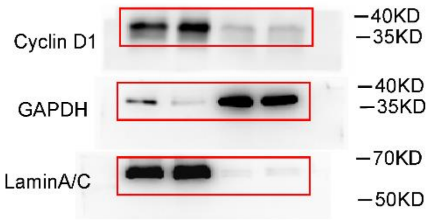

Fig. 4f

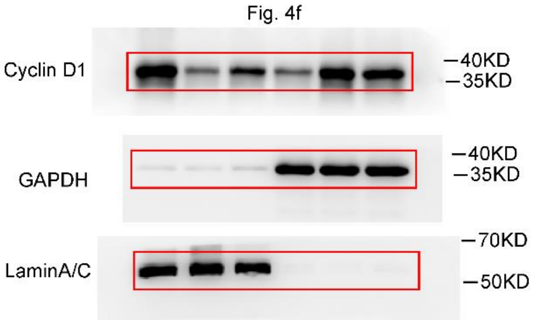

Fig. 4g

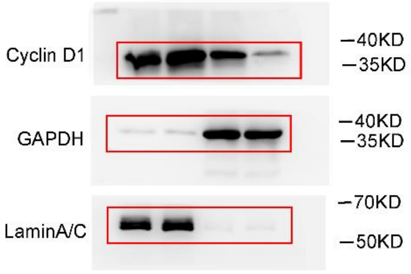

Fig. 4h

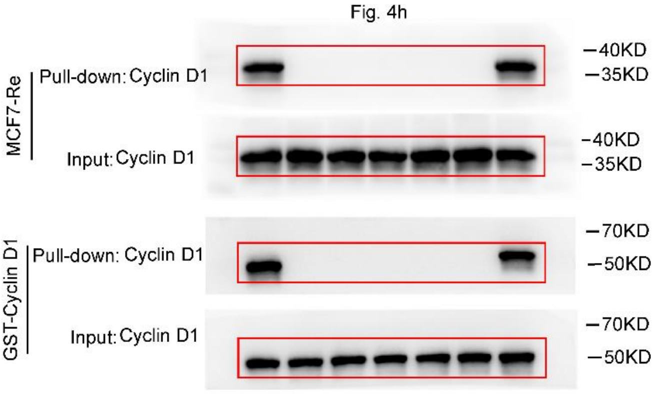

Fig. 4j

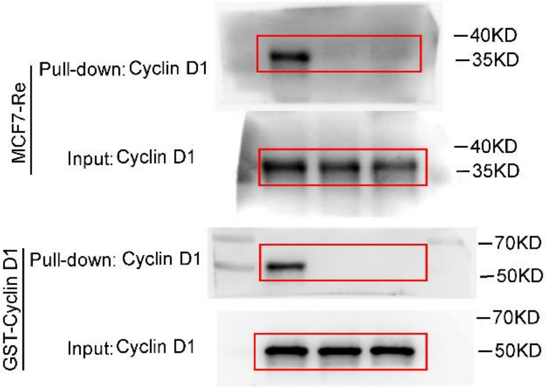

Fig. 4k

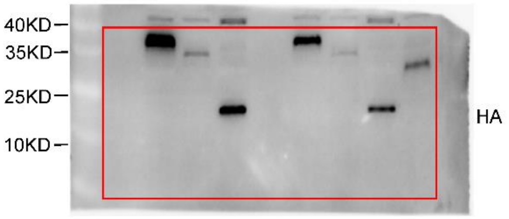

Fig. 4l

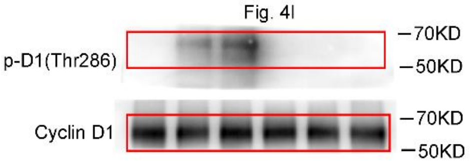

Fig. 4o

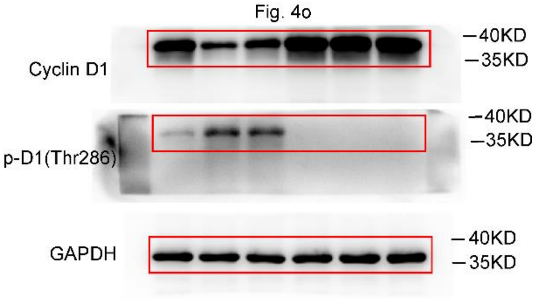

Fig. S1e

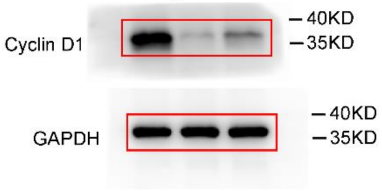

Fig. S1f

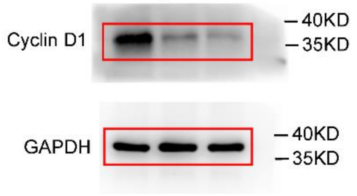

Fig. S2f

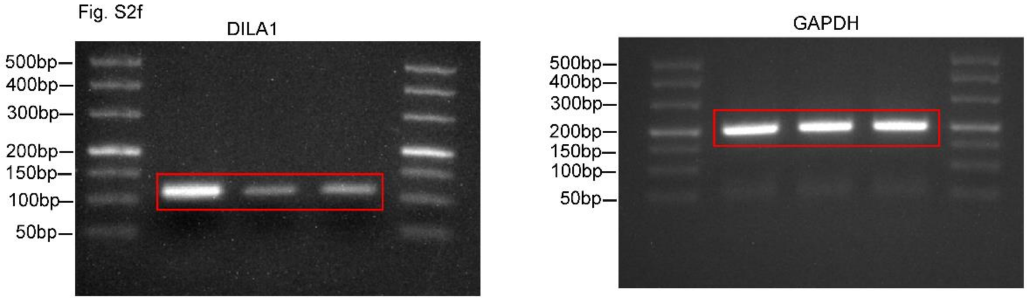

Fig. S5a

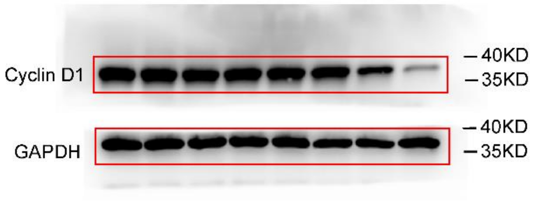

Fig. S5b

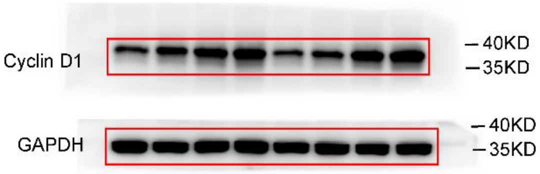

Fig. S5e

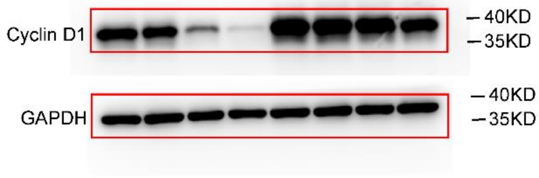

Fig. S5g

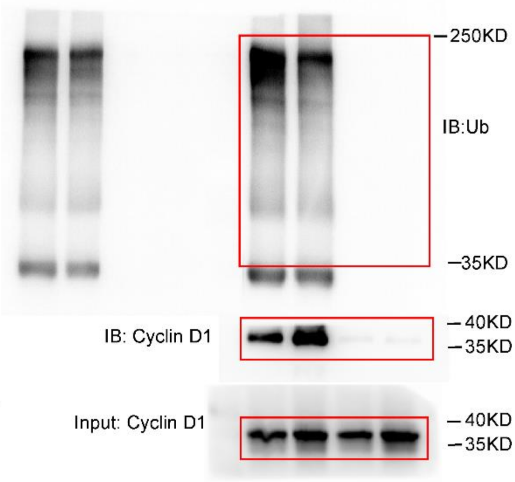

Fig. S5f

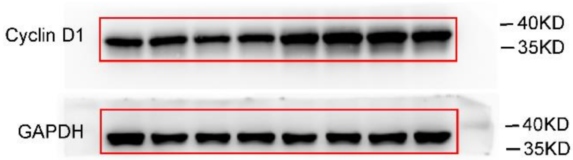

Fig. S6a

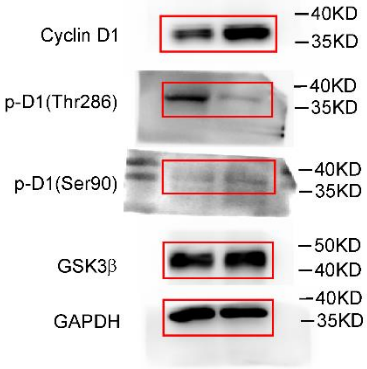

Fig. S6b

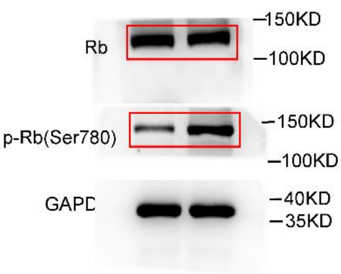

Fig. S6c

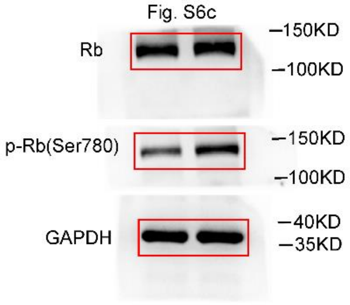

Fig. S6d

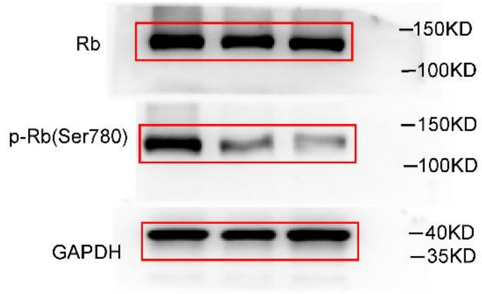

Fig. S6e

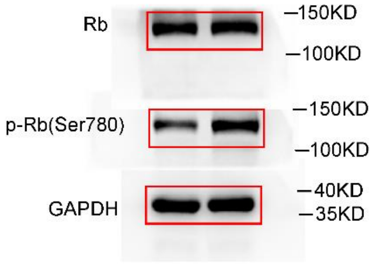

Fig. S7c

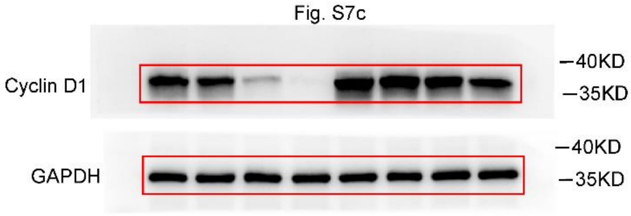

Fig. S7d

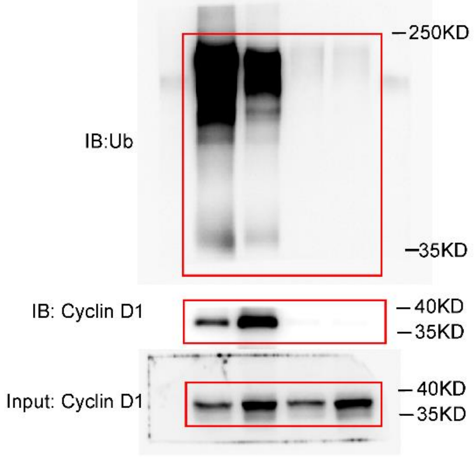

Fig. S7e

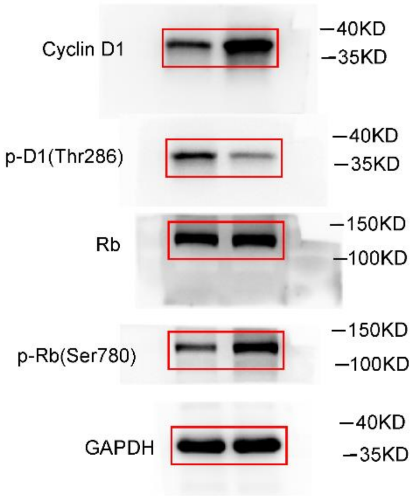

Fig. S7f

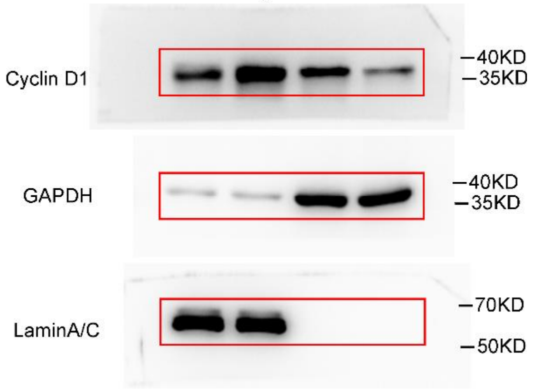

Supplement: Supplementary file 1 — Supplementary Information [file 41467_2020_19349_MOESM1_ESM.pdf]
